# MnCl_2_(C_6_N_10_H_6_): Insights into a Luminescent Transition Metal–Melem Complex

**DOI:** 10.3390/molecules29235598

**Published:** 2024-11-27

**Authors:** Elaheh Bayat, Markus Ströbele, David Enseling, Thomas Jüstel, Hans-Jürgen Meyer

**Affiliations:** 1 Section for Solid State and Theoretical Inorganic Chemistry, Institute of Inorganic Chemistry, University of Tübingen, Auf der Morgenstelle 18, 72076 Tübingen, Germany; elaheh.bayat@anorg.uni-tuebingen.de (E.B.); markus.stroebele@uni-tuebingen.de (M.S.); 2 Department of Chemical Engineering, FH Münster University of Applied Sciences, Stegerwaldstraße 39, 48565 Steinfurt, Germany; david.enseling@fh-muenster.de (D.E.); juestel@fh-muenster.de (T.J.)

**Keywords:** melem, melamine, coordination sites of melem, manganese chloride, photoluminescence, transition metal carbide

## Abstract

In this work, the (MnCl_2_(C_6_N_10_H_6_) complex has been synthesized via solid-state reaction between manganese (II) chloride and melamine in the molar ratio of 1:2. A similar synthesis has been repeated with CoCl_2_, and FeCl_2_, resulting in two new metal–melam complexes (FeCl_2_(C_6_N_11_H_9_) and CoCl_2_(C_6_N_11_H_9_)). MnCl_2_(C_6_N_10_H_6_) crystallizes in the monoclinic crystal system with the space group *I*2/*a*. The crystalline powder of MnCl_2_(C_6_N_10_H_6_) was studied by X-ray diffraction, infrared spectroscopy, and thermogravimetric analysis to examine its structure and properties. MnCl_2_(C_6_N_10_H_6_) also shows good thermal stability up to 370 °C; however, the complete decomposition occurred at 900 °C, yielding Mn_7_C_3_. This paper presents an easy synthesis of the first luminescent transition metal–melem complex, providing new insights into the reactivity of melamine at elevated temperatures in the presence of transition metal chlorides.

## 1. Introduction

The first synthesis of melamine (1,3,5-triazine-2,4,6-triamine) dates back to a century ago with the reaction of thiocyanate with ammonium chloride [1]. Later on, there were more synthetic methods suggested by many researchers to yield melamine based on heating thiourea, guanidine carbonate, cyanamide, or dicyandiamide [1,2]. Nowadays, urea is a precursor for the industrial production of melamine, which has increased the production of melamine to millions of tons per year, making this material widely available.

**Figure 1:**
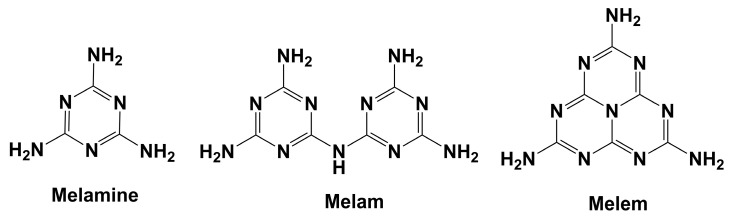
Structures of melamine (C_3_N_6_H_6_), melam (C_6_N_11_H_9_) and melem (C_6_N_10_H_6_).

When pure melamine (C_3_N_6_H_6_) is heated up, it will form different condensation products such as melam (C_6_N_11_H_9_) at around 360 °C [3] (340 °C [4]), and subsequently melem (C_6_N_10_H_6_) at approximately 400 °C [3] (380 °C [4]) (Figure 1). This process is accompanied by the release of ammonia during the condensation. Interestingly, during this transformation process, the characteristic rings of triazine (cyanuric nuclei) and heptazine (cyameluric nuclei) are retained or restructured. Triazine rings are composed of a single six-membered ring with alternating carbon and nitrogen atoms, as can be found in melamine. Heptazine rings, on the other hand, consist of three fused triazine rings which create a larger and more complex structure of melem and melon [5].

The formation of extended supramolecular structures based on the molecular entities of melem and melon through the thermal condensation of melamine is a conventional way to form metal-free molecules and polymers [6]. Derivatives of s-heptazine are particularly interesting due to their intriguing thermal stability and unique electronic structures. The formation of ionic and polymeric carbon nitride compounds [7,8,9] based on aromatic tricyclic units (tri-s-triazine, C_6_N_7_) typically involves an ordered self-assembly with bonding via covalent and noncovalent interactions [2]. These polymeric materials are represented by an extended network of melem, connected by hydrogen bonding and π-π stacking, featuring surprisingly high thermal stabilities. The research on carbon nitride compounds is extensive, covering a wide range of materials. Depending on the bonding arrangement and ratio of carbon to nitrogen atoms, these compounds are classified with their very own nomenclature, such as triazine-based polymers [10], graphitic carbon nitride, carbon nitride nanotubes, boron carbon nitrides, and so on. Melem and carbon nitride (C_3_N_4_) were reported for their potential applications in flame retardance [11], photocatalysis [12,13], heterogeneous catalysis [14], as nanosheets for bioimaging [15], luminescence devices [16], the anode material of lithium-ion battery [17], and as an adsorbent of heavy metals and dyes [18,19], etc.

Carbon-nitride materials have significant and various applications; however, there are few studies on the reactivity of melem up to now [12,20]. Early studies on s-heptazine derivatives faced challenges because of the insolubility (in water and any organic solvents) and low reactivity of melem [21]. S-heptazine and s-triazine are considered electron-deficient aromatic compounds due to the existence of nitrogen atoms in the ring, which makes them able to undergo nucleophilic substitution under specific conditions [20]. Yet, the chemical behavior of melem remains less explored when compared to the triazine analog. There are only limited examples of the reactivity of NH_2_-groups of melem reported up to now [12,20]. Examples of such studies include those on 2,5,8-triphthalimido-tri-s-triazine [22], 2,5,8-tri(2,3,4,5-tetrafluorophthalimido)-tri-s-triazine [23]. Furthermore, by treating melem with mineral acids, several melemium salts were obtained such as phosphate salt C_6_N_7_(NH_2_)_3_·H_3_PO_4_, sulfate salt H_2_C_6_N_7_(NH_2_)_3_SO_4_·2H_2_O, melemium melem perchlorate HC_6_N_7_(NH_2_)_3_ClO_4_·C_6_N_7_(NH_2_)_3_, etc. [24,25].

Recent research by Xu et al. has explored the reactivity of melem with various metals. Their study involved the interactions of melem in an aqueous suspension with AgNO_3_, Zn(NO_3_)_2_, Cu(NO_3_)_2_, Co(NO_3_)_2_, and Ni(NO_3_)_2_. Among these, the only compound obtained was an infinite Ag-N nanocage coordinated with melem [26]. Simultaneously, our research group investigated the formation of complex metal-halide–melem compounds by reacting binary metal halides with melem, which introduces compounds of CaBr_2_, SrBr_2_, SrI_2_, BaI_2_, and PbBr_2_ with melem [27].

In this study, we explored the solid-state reactivity of melamine in the presence of transition metal chlorides at higher temperatures, where melam and melem can form new complexes of FeCl_2_(C_6_N_11_H_9_), CoCl_2_(C_6_N_11_H_9_), and more importantly MnCl_2_(C_6_N_10_H_6_). MnCl_2_(C_6_N_10_H_6_) has been characterized by powder X-ray diffraction (PXRD), single-crystal diffraction, and infrared (IR) spectroscopy. Additionally, thermogravimetric analyses (TGA) were conducted to evaluate the stability of MnCl_2_(C_6_N_10_H_6_) and to determine its decomposition products. The TGA analysis indicates that manganese carbodiimide [28] is formed as an intermediate compound at 700 °C, and at higher temperatures, the decomposition product is manganese carbide, Mn_7_C_3_ (ICSD 69534).

This transition metal carbide has been previously synthesized in the carbothermal reduction of manganese oxide in two steps, forming MnO at 1050 °C and Mn_7_C_3_ at 1300 °C. Alternatively, Mn_7_C_3_ can also be obtained from the reaction of manganese dust with n-pentane at 850 °C at reduced pressure [29]. Additionally, various researchers have proposed other methods for synthesizing this carbide [30]. Due to the wide range of applications of transition metal carbides in the heat-resistance and hard material industry, Mn_7_C_3_ is valued [31]. Furthermore, manganese carbodiimide and manganese carbide can exhibit various applications due to the position of manganese in the middle of the 3d series [30].

Finally, the photoluminescence properties of MnCl_2_(C_6_N_10_H_6_) are discussed, providing comprehensive insight into its remarkable photochemical behavior

## 2. Results and Discussion

### 2.1. Crystal Structure of MnCl_2_(C_6_N_10_H_6_), and FeCl_2_(C_6_N_11_H_9_)

**Figure 2:**
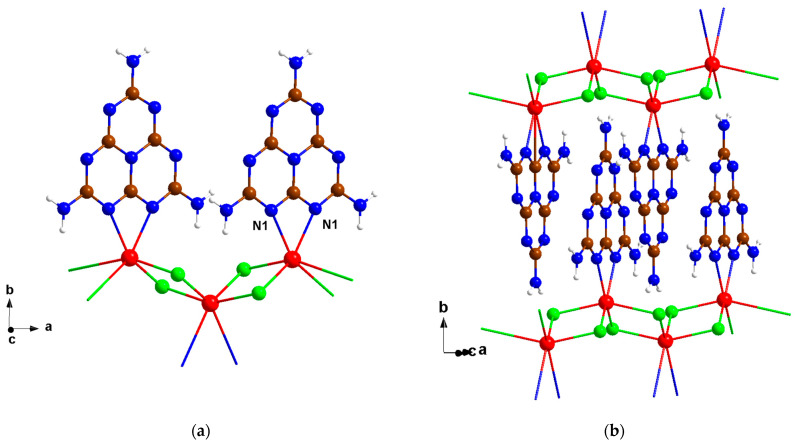
(**a**) A perspective view of the crystal structure of MnCl_2_(C_6_N_10_H_6_) of the *ab*-plane and (**b**) the stacking along the *b-*axis (with the color code for N: blue, C: brown, H: white, Cl: green, and Mn: red).

The MnCl_2_(C_6_N_10_H_6_) crystallizes in a monoclinic crystal system and the space group of *I*2/*a* with crystallographic details summarized in Table 1. The crystal structure is composed of MnCl_4/4_ chains along the *a*-axis that are interconnected by melem units to form a layered arrangement (Figure 2). The melem units are connected to the manganese atom through the two inner nitrogen atoms of N1, with a bond distance of 2.331 (5) Å, thereby completing the coordination number six of manganese. These melem units are situated between the MnCl_4_/_4_ layers, as shown in Figure 2b. FeCl_2_(C_6_N_11_H_9_) crystallizes in a monoclinic crystal system, in the space group P21/c. The iron chloride is coordinated through N1 and N7 to the bidentate melamine ligand binding a melam unit. The steric demand and tetrahedral environment of the central atom force the melamine ligand to protrude from the plane, disrupting the planarity of the triazine rings. The crystal structure of FeCl_2_(C_6_N_11_H_9_), along with the crystallographic details, is presented in Figure S1 and Table S1, respectively.

Crystals of CoCl_2_(C_6_N_11_H_9_) were obtained under the same reaction conditions. The PXRD pattern closely matches the calculated pattern based on the single-crystal refinement of FeCl_2_(C_6_N_11_H_9_), indicating both structures to be isotypic.

### 2.2. Thermoanalytic Studies

**Figure 3:**
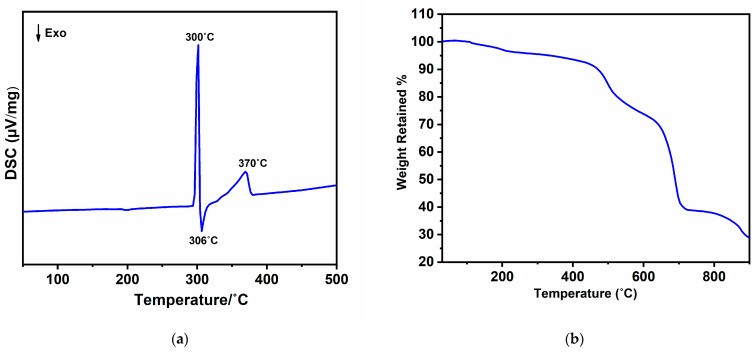
(**a**) DSC of the reaction of MnCl_2_ and melamine in a molar ratio of 1:2 and (**b**) TGA of MnCl_2_(C_6_N_10_H_6_).

A valuable technique that can provide insights into the formation or decomposition of new phases is combining differential scanning calorimetry (DSC) with X-ray diffraction (XRD). In the DSC analysis of a 1:2 molar mixture of manganese (II) chloride and melamine, as shown in Figure 3a, there are three notable thermal effects: two endothermic peaks centered at 300 °C and 370 °C, and an exothermic peak at 306 °C. The endothermic peak at 300 °C is followed by an exothermic peak at 306 °C, which is attributed to the formation of an unknown intermediate phase (see Figure S2), which subsequently decomposes around 370 °C, resulting in the formation of MnCl_2_(C_6_N_10_H_6_). The stability of MnCl_2_(C_6_N_10_H_6_) has been further investigated using thermogravimetric analysis (TGA).

The compound demonstrates good thermal stability up to 400 °C, with only a 2.6% weight loss. This reduction in weight may be attributed to a small amount of an amorphous side phase from the reaction, which will be further explained in the next section. The TGA results, presented in Figure 3b, indicate that the complex gradually decomposes into different compounds when it is heated to 900 °C. To better understand the decomposition process, the TGA was repeated, with the analysis stopped at specific temperature intervals (approximately 500 °C, 700 °C, and 900 °C) to identify the decomposition products. At around 500 °C, an ex situ powder X-ray diffraction (PXRD) analysis revealed that MnCl_2_(C_6_N_10_H_6_) had decomposed into an amorphous intermediate phase. By 700 °C, the complex forms manganese carbodiimide [28] (Figure S3a). Finally, at 900 °C, the complex underwent complete decomposition, resulting in the formation of the transition metal carbide Mn_7_C_3_, as confirmed by the XRD pattern shown in Figure S3b.

### 2.3. X-Ray Powder Diffraction

**Figure 4:**
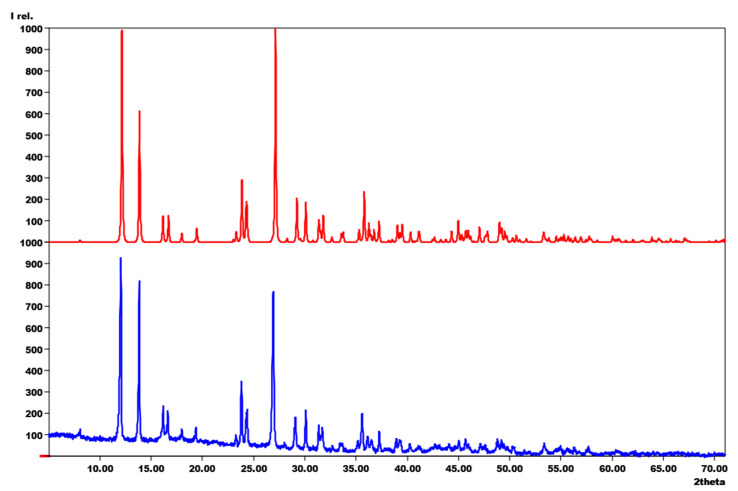
XRD powder pattern of the synthesized MnCl_2_(C_6_N_10_H_6_) (bottom), compared with the calculated pattern based on the single-crystal refinement (top) (CCDC code: 2141509).

The reaction between MnCl_2_ and melamine in the ratio of 1:2 at 400 °C for 100 h yields a product where MnCl_2_(C_6_N_10_H_6_) can be isolated as a separate phase. From the theoretical Equation (1), we would have expected that this product is formed by the release of NH_3_ and can be obtained as a pure phase (Figure 4). However, the XRD powder pattern showed a high background after the first synthesis, implying that there might be an amorphous phase. Therefore, in order to purify the MnCl_2_(C_6_N_10_H_6_), a double chamber ampule with a temperature gradient (Figure S4) was utilized. Subsequently, the product (orange powder, Figure S4) is subjected to analysis via powder X-ray diffraction (PXRD), with the resulting diffractogram then compared with the calculated pattern derived from structure refinement based on single-crystal data (Figure 3). The white residue on the colder side of the ampule has been also analyzed, which shows that the amorphous phase is crystallized into melamine and ammonium chloride (Figure S4a,b).

MnCl_2_ + 2C_3_N_6_H_6_ → MnCl_2_(C_6_N_10_H_6_) + 2NH_3_ (1)

Figure S5 presents the PXRD patterns of two complexes of FeCl_2_(C_6_N_11_H_9_) and CoCl_2_(C_6_N_11_H_9_). Similarly, the high background of the XRD pattern can be attributed to either the fluorescence effect of Fe and Co or the presence of an amorphous phase. These complexes were also prepared from a molar ratio of 1:2 of FeCl_2_, and CoCl_2_ with melamine, in the same condition as a synthesis of MnCl_2_(C_6_N_10_H_6_). The theoretical reaction equation is shown in Equations (2), and (3). These complexes closely resemble those previously introduced by our group, including LiBr(C_6_N_11_H_9_) (CCDC: 2039843), CuX(Cl,Br,I)(C_6_N_11_H_9_) (CCDC: 2057533, 2059117, 2041008, respectively), and ZnI_2_(C_6_N_11_H_9_) (CCDC: 2056462).

FeCl_2_ + 2C_3_N_6_H_6_ → FeCl_2_(C_6_N_11_H_9_) + NH_3_ (2)

CoCl_2_ + 2C_3_N_6_H_6_ → CoCl_2_(C_6_N_11_H_9_) + NH_3_ (3)

### 2.4. Infrared (IR) Spectroscopy

**Figure 5:**
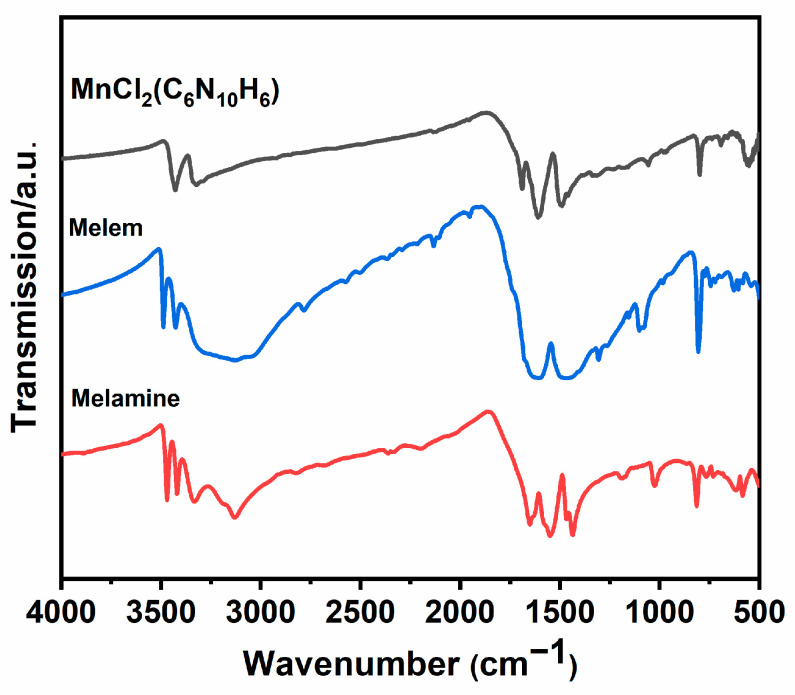
FT-IR-spectrum of MnCl_2_(C_6_N_10_H_6_) compared to melamine and melem.

The IR spectrum of the MnCl_2_(C_6_N_10_H_6_) complex was compared with the spectra of melamine and melem as shown in Figure 5. Table S2 shows details of the frequencies related to the different vibrational modes of these compounds and the corresponding bond assignments. As illustrated in Figure 5, infrared spectra for all three compounds were recorded in the range from 4000 to 500 cm^−1^. This comparison aids in visualizing the similarity and difference of the MnCl_2_(C_6_N_10_H_6_) with that of melem and linking them to the established vibrational modes. As expected, because all the compounds contain NH_2_ groups, similar spectral patterns in the 3500–3100 cm^−1^ and 1580–1600 cm^−1^ regions are related to NH stretching and bending vibrations. However, MnCl_2_(C_6_N_10_H_6_) reveals notable differences from melamine in these areas. Despite some minor changes in intensity and splitting, the vibrational features of MnCl_2_(C_6_N_10_H_6_) are similar to those of melem.

### 2.5. Photoluminescence Measurements

The photoluminescence (PL) properties of Mn(II) complexes, whether in inorganic compounds [32] or organic–inorganic complexes [33,34,35], are particularly captivating. These complexes exhibit intriguing optical properties and have potential applications in sensors, optical devices, optical markers, and cost-effective OLEDs [33]. The PL of Mn^2^⁺ is primarily associated with 3d-3d transitions [36,37]. However, its photoluminescence can be significantly affected by both the local coordination environment of the manganese ions and the overall crystal structure. Mn^2+^ comprising luminescent materials with multiple 3d-3d transitions (see Figure S6, Tanabe-Sugano-Diagram for d^5^ ions) have been known since the forties of the last century [38], while the Mn-centered absorption lines are rather weak due to the spin and Laporte forbidden character of these 3d-3d transitions. Therefore, applied Mn^2+^ luminophores are sensitized, either by the band-to-band transition of the host, e.g., in the widely applied EL and display phosphors ZnS:Mn or Zn_2_SiO_4_:Mn [39,40]. Alternatively, sensitization is achieved by a co-activator, as in the fluorescent lamp phosphors Ca_5_(PO_4_)_3_(F,Cl):Sb,Mn and BaMgAl_10_O_17_:Eu,Mn [41,42], or by ligand-centered transitions as in coordination compounds. In its pure phase, MnCl_2_ displays different emission characteristics under varying pressures [43]. At ambient pressure, the emission occurs at 642 nm (15,580 cm^−1^), attributed to the spin-forbidden ^4^T_1g_(G) → ^6^A_1g_(^6^S) transition [36,37,43]. As pressure increases, a red-shift of the emission band is observed as the ligand to metal distances decline and the crystal field strength increases. Moreover, the color of the emission from Mn(II) complexes and phosphors is dependent on the coordination geometry. In a tetrahedral field, the emission is typically green, while in an octahedral crystal field, it is observed from the red to the orange range [43].

**Figure 6:**
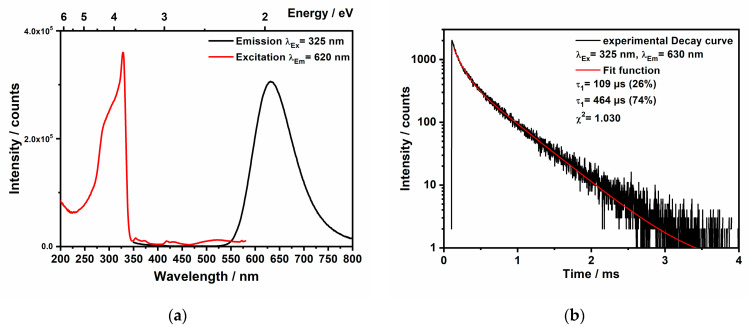
(**a**) Photoluminescence spectra of MnCl_2_(C_6_N_10_H_6_) in the solid-state at room temperature. (**b**) Decay curve obtained for MnCl_2_(C_6_H_6_N_10_) upon 325 nm excitation at room temperature.

In this study, MnCl_2_(C_6_N_10_H_6_) exhibits red photoluminescence upon excitation with a UV radiation source. The emission spectrum reveals a broad band centered at 620 nm (16,130 cm^−1^), while the FWHM is 2460 cm^−1^. Weak excitation bands are observed between 350 and 500 nm (Figure 6), which are attributed to the Mn^2+^-centered transitions between the ground state term ^6^A_1g_ and the excited state terms 4T_1g_, 4T_2g_, 4A_1g_, and ^4^E_g_ [41]. The strong excitation band at 325 nm is caused by the coordinated ligand melem, which sensitizes the Mn^2+^ luminescence.

The decay measurement after a 325 nm excitation delivers a biexponential decay curve with an emission lifetime τ_1_ of 109 μs (26%) and a longer component with τ_2_ of 464 μs (74%). The decay time of Mn^2+^-activated phosphors with a high quantum yield close to unity is in the range of 8 to 40 ms [44], while the decay time of Mn^2+^ in coordination compounds is in the range from 0.1 to 25 ms [33]. However, the decay time can be strongly reduced by concentration quenching, by Mn^2+^ ions at the particle surface [45], and/or by magnetic interaction (superexchange) between ligand-bridged Mn^2+^ ions [46]. We observed a low quantum yield < 10%, which is in line with a rather short decay time. Upon comparing the qualitative absorption spectrum of MnCl_2_(C_6_N_10_H_6_) (Figure S7) with the UV/Vis absorption spectra of melem [6,47], it is evident that while the absorption in pure melem completely diminishes in the visible-light region, the absorption in MnCl_2_(C_6_N_10_H_6_) shows a similar initial decline but persists slightly longer, though only at a very low intensity (approximately 2% of the absorption), before fully fading away at a wavelength of 600 nm. Melem has also been previously coupled with other organic monomers to construct extended conjugated networks, thereby enhancing visible-light absorption and improving photocatalytic performance [6].

## 3. Materials and Methods

The starting materials, MnCl_2_ (ABCR, Nagano, Japan, 97%), FeCl_2_ (ABCR, 98%) and CoCl_2_ (ABCR, 97%), and melamine (Sigma-Aldrich, St. Louis, MO, USA, 99%), were used as received without additional purification. All the handling and storage of these materials were conducted within a glovebox, maintaining an argon atmosphere with moisture and oxygen levels below 1 ppm.

For the synthesis of MnCl_2_(C_6_N_10_H_6_), the reaction mixture was prepared with a molar ratio of 1:2 for MnCl_2_ to melamine. This mixture was then transferred into a hand-made silica tube with a length of 6 cm, an outer diameter of 10 mm, and an inner diameter of 7 mm. The resulting mixture, weighing approximately 50 g, was vacuum-sealed. This ampule was then placed in a Carbolite furnace, where it was heated at a rate of 1 °C min^−1^ to 400 °C and remained at this temperature for 100 h, followed by cooling at a rate of 0.1 °C min^−1^ to room temperature. The crystals of MnCl_2_(C_6_N_10_H_6_) appeared on the wall of the ampule slightly above the crystalline powder. To prepare the crystalline powder of MnCl_2_(C_6_N_10_H_6_), the reaction time can be reduced to 20 h with a heating and cooling rate of 1 °C min^−1^. The obtained XRD powder pattern revealed the presence of small unidentified peaks and a high background, suggesting the presence of an unknown amorphous phase. To purify the sample, a double chamber ampule, as shown in Figure S4, was used. One chamber of ampule was placed in a glass oven at 350 °C for 72 h, while the other chamber was outside of the furnace at room temperature. The pure phase remained on the hot side of the ampule, while the side phase, consisting of ammonium chloride and melamine, was separated. The yield of the reaction is estimated to be around 64%.

FeCl_2_(C_6_N_11_H_9_) and CoCl_2_(C_6_N_11_H_9_) were synthesized in a similar route by mixing one molar ratio of FeCl_2_, or CoCl_2_ with 2 molar ratios of melamine (Sigma-Aldrich, 99%). A total of 50 mg of each mixture was transferred into a 6 cm ampule and heated to 400 °C in the Carbolite furnace for 100 h (with a ramp of 1 and 0.1 °C min^−1^). To obtain crystals of these complexes, the same mixture was subjected to the same conditions except in a Simon furnace with a very small temperature gradient. At the bottom of the ampule, we could see a few crystals of FeCl_2_(C_6_N_11_H_9_) and CoCl_2_(C_6_N_11_H_9_). (If the temperature gradient is too high, the formation of ammonium melem chloride hinders the formation of main phases). The reaction scheme of all three compounds is shown in Figure S8.

X-ray diffraction patterns of the prepared powders were recorded using a powder diffractometer (STOE, Darmstadt, Germany, STADIP, Ge-monochromator) with Cu-Kα_1_ radiation (λ = 1.540598 Å). Data were collected in the range of 5 < 2θ < 70°. The patterns were then compared to those of the relevant crystal structures using Match3! Software [48].

Single crystals of MnCl_2_(C_6_N_10_H_6_) were selected and mounted on a Rigaku XtaLab Synergy-S single-crystal X-ray diffractometer [49]. X-ray diffraction data were collected using Cu-Kα radiation (λ = 1.54184 Å) and a mirror monochromator, with measurements taken at a temperature of 180 K. Crystal structures were determined using direct methods (SHELXT), followed by full-matrix least-squares refinement (SHELXL-2014) [50,51]. X-ray intensity absorption corrections were applied using numerical methods with CrysAlisPro 1.171.41.92a software (Rigaku Oxford Diffraction, Neu-Isenburg, Germany) [49]. Hydrogen atoms were identified in the difference maps and refined isotropically based on their positions. The crystal structure of FeCl_2_(C_6_N_11_H_9_) was also solved and refined on the basis of single-crystal X-ray diffraction data (Table S1).

Differential scanning calorimetry (DSC) was performed using a DSC 204 F1 Phoenix instrument (Netzsch, Selb, Germany). In a glovebox, under an argon atmosphere, the starting materials were sealed in 100 μL gold-plated (5 μm) steel autoclaves (Bächler Feintech AG, Hölstein, Switzerland). The reaction between MnCl_2_ and melamine, with a 1:2 ratio, was investigated over a temperature range from room temperature to 500 °C, applying heating and cooling rates of 2 and 0.5 °C/min.

For thermogravimetric analysis (TGA), a Netzsch Jupiter STA 449 F3 apparatus was employed. The final product was placed in a hand-made open-ended silica container under argon and underwent gradual heating and cooling at a rate of 2 K/min. This approach allowed for the evaluation of the product’s thermal stability across a temperature range from room temperature to 900 °C.

Infrared (IR) spectra of the samples were acquired using a Bruker (Frankfurt, Germany) VERTEX 70 FT-IR spectrometer, covering the spectral range from 400 to 4000 cm^−1^. KBr tablets were utilized as a background reference.

For optical measurements the emission and excitation spectra of MnCl_2_(C_6_N_10_H_6_) were recorded optically using the fluorescence spectrometer FLS920 (Edinburgh Instruments, Livingston, UK) equipped with a 450 W xenon discharge lamp (Osram, Munich, Germany). A mirror optic designed for powder samples was also utilized. An R2658P single-photon-counting photomultiplier tube manufactured by Hamamatsu was used for detection. Photoluminescence spectra were recorded with a spectral resolution of 1 nm, a dwell time of 0.5 s at 1 nm intervals, and 2 repetitions. Photoluminescence decay curves were measured using the same spectrometer, with a 445 nm picosecond laser serving as the pulsed excitation source.

## 4. Conclusions

In conclusion, the successful synthesis of MnCl_2_(C_6_N_10_H_6_) is a very important step toward understanding the coordination chemistry of melem. We attempted to extend this work by investigating the solid-state reactivity of melamine with transition metal chlorides at elevated temperatures. This approach led to the successful synthesis of several new coordination complexes with melam, such as FeCl_2_(C_6_N_11_H_9_), and CoCl_2_(C_6_N_11_H_9_).

It is noteworthy to mention that our primary focus is on synthesizing MnCl_2_(C_6_N_10_H_6_) since it is the first luminescent transition metal–melem complex. Infrared (IR) spectroscopy, powder X-ray diffraction (PXRD), and single-crystal X-ray diffraction were used to characterize the structure of MnCl_2_(C_6_N_10_H_6_). Thermal gravimetric analysis (TGA) also provided insights into the thermal stability and decomposition of this compound, showing that MnCl_2_(C_6_N_10_H_6_) is first decomposed into manganese carbodiimide [28] at 700 °C and then at 900 °C to manganese carbide (Mn_7_C_3_). The synthesis of manganese carbide, which is typically produced via carbothermal reduction processes, has applications in heat-resistant and hard materials. Proposing a new synthetic route for manganese carbodiimide and Mn_7_C_3_ is also another aspect of this work. Finally, the photoluminescence properties of MnCl_2_(C_6_N_10_H_6_) were studied which shows red-to-orange fluorescence with an emission peak at 620 nm and a biexponential decay with a lifetime in the 100 µs range.

## Figures and Tables

**Table 1:** Crystallographic details of the crystal structure refinement of MnCl_2_(C_6_N_10_H_6_).

Empirical Formula	MnCl_2_(C_6_N_10_H_6_)	
CCDC code	2141509	
Formula weight (g/mol)	344.05	
Wavelength (Å)	1.54184	
Crystal system	Monoclinic	
Space group	*I* 1 2/*a* 1	
Unit cell dimensions (Å)	*a*/Å	6.6697 (4)
	*b*/Å	21.926 (1)
	*c*/Å	7.718 (2)
Volume (Å^3^)	1128.61	
*Z*	4	
Density (calculated) (g/cm^3^)	2.025	
Absorption coefficient (mm^−1^)	13.947	
Final R indices (I > 2σ(I))	*R*1 = 0.0288, wR2 = 0.0581	
R indices (all data)	*R*1 = 0.0333, wR2 = 0.0594	
GOOF	1.070	

## Data Availability

Data are contained within the article and Supplementary Materials.

## Supplementary Materials

The following supporting information can be downloaded at: https://www.mdpi.com/article/10.3390/molecules29235598/s1, Figure S1: Section of the crystal structure of FeCl_2_(C_6_N_11_H_9_) projected on *bc*-plane (top) and the unit cell content of the structure (bottom). Table S1: Crystallographic details of the crystal structure refinement on FeCl_2_(C_6_N_11_H_9_).; Figure S2: Ex-situ powder XRD pattern of an unknown phase formed by heating a mixture of MnCl_2_ and melamine in a 1:2 ratio to the first exothermic DSC peak, observed at 306 °C. Figure S3: a. XRD pattern of manganese carbodiimide (MnCN_2_) obtained by heating MnCl_2_(C_6_H_6_N_10_) to 700 °C, along with reflections of unknown side-phase (shown with black stars) compared with the calculated pattern based on the single-crystal structure refinement (top) (CCDC code: 272236). b. XRD pattern of manganese carbide (Mn_7_C_3_) obtained from heating MnCl_2_(C_6_N_10_H_6_) to 900 °C (bottom), compared with the calculated pattern based on the single-crystal refinement (top) (CCDC code: 2141509). Figure S4: a. Photograph of a two-chamber ampule used for separation of side-phase from MnCl_2_(C_6_N_10_H_6_) under daylight (top) and under UV irradiation (bottom). (The observed blue light observed in the Figure originates from the reflectance of the blue light (366 nm) on the white powder. b. XRD pattern of side phase on the left side of two-sided chamber. Figure S5: Recorded XRD patterns of FeCl_2_(C_6_N_11_H_9_), CoCl_2_(C_6_N_11_H_9_) with the calculated pattern from the structure refinement of FeCl_2_(C_6_N_11_H_9_) (top). Table S2: Vibrational frequencies (in cm^−1^) for MnCl_2_(C_6_N_10_H_6_) compared to those of melamine and melem. Figure S6: Tanabe-Sugano-Diagram for a d^5^ ion with the most prominent emission transition between ^4^T_1_(^4^G) and ^6^A_1_(^6^S). Figure S7: A qualitative absorption spectrum by the aid of the Kubelk-Munk function from the reflection spectrum of MnCl_2_(C_6_N_10_H_6_). Figure S8: A reaction scheme of melamine with some transition metal chlorides to obtain FeCl_2_(C_6_N_11_H_9_), CoCl_2_(C_6_N_11_H_9_), and MnCl_2_(C_6_N_10_H_6_) compounds.
