# Supplementary material for: MnCl2(C6N10H6): Insights into a Luminescent Transition Metal–Melem Complex
Source: Molecules. 2024 Nov 27;29(23):5598. doi: 10.3390/molecules29235598 (PMC11643902; doi:10.3390/molecules29235598)
Supplement: Supplementary file 1 [file molecules-29-05598-s001.zip › molecules-3264041-supplementary.pdf]

$MnCl_2(C_6N_{10}H_6)$: Insights into a Luminescent Transition Metal–Melem Complex

Elaheh Bayat [1], Markus Ströbele [1], David Enseling [2], Thomas Jüstel [2] and Hans-Jürgen Meyer [1,*]

[1] Section for Solid State and Theoretical Inorganic Chemistry, Institute of Inorganic Chemistry, University of Tübingen, Auf der Morgenstelle 18, 72076 Tübingen, Germany

[2] Department of Chemical Engineering, FH Münster University of Applied Sciences, Stegerwaldstraße 39, 48565 Steinfurt, Germany

***** Correspondence: juergen.meyer@uni-tuebingen.de

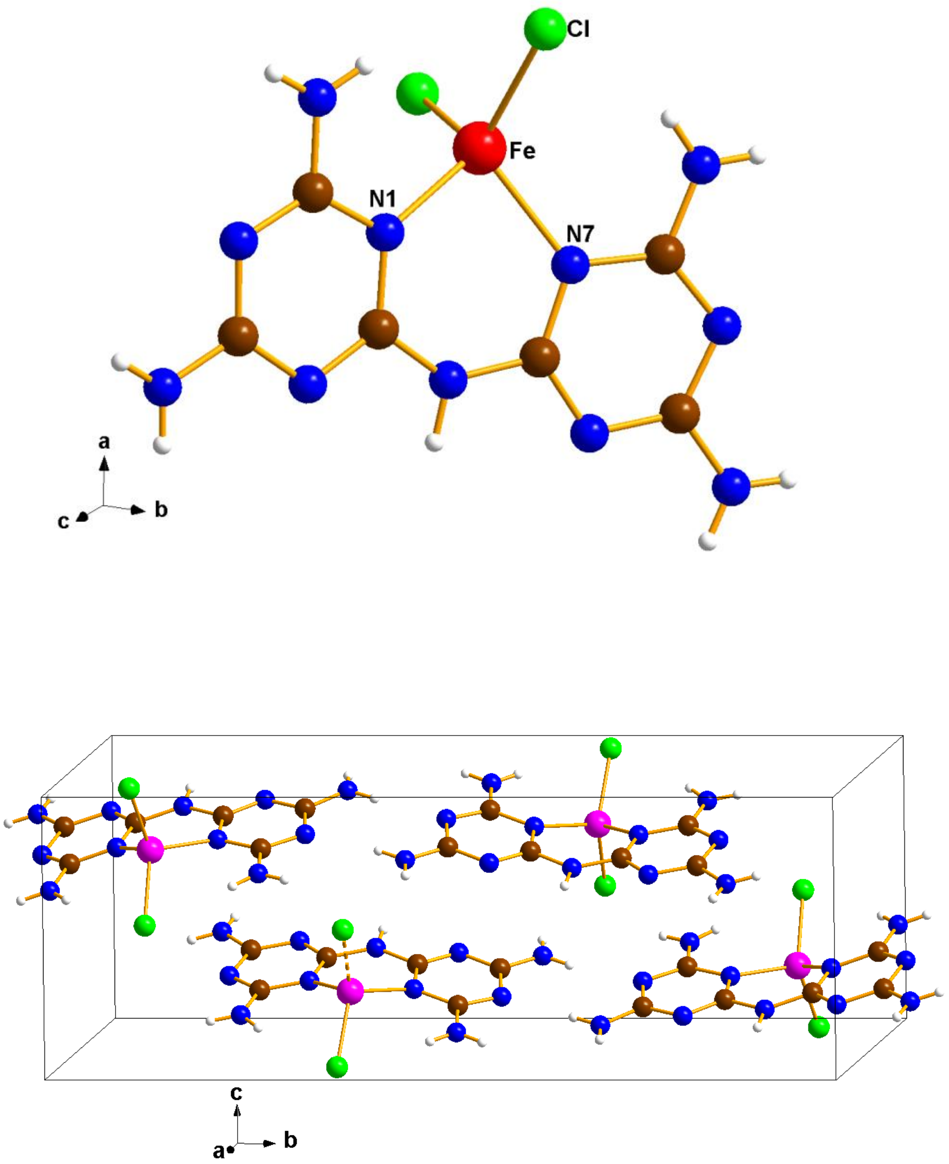

Figure S1. Section of the crystal structure of $FeCl_2(C_6N_{11}H_9)$ projected on *bc*-plane (top) and the unit cell content of the structure (bottom).

Table S1. Crystallographic details of the crystal structure refinement on $FeCl_2(C_6N_{11}H_9)$.

**Empirical Formula**	**$FeCl_2(C_6N_{11}H_9)$**	
CCDC code	2159999	
Formula weight (g/mol)	361.99	
Wavelength (Å)	1.54184	
Crystal system	Monoclinic	
Space group	$P\ 1\ 2_1/c\ 1$	
Unit cell dimensions (Å)	*a*/Å	7.472(6)
	b/Å	22.29(3)
	c/Å	7.693(1)
Volume ($Å^3$)	1261.40(3)	
Z	4	
Density (calculated) (g/cm^3)	1.906	
Absorption coefficient (mm^{-1})	13.613	
Final R indices ($I>2\sigma(I)$) a)	*R*1 = 0.0208, wR2 = 0.0584	
R indices (all data)	*R*1 = 0.0217, wR2 = 0.0589	
GOOF	1.083	

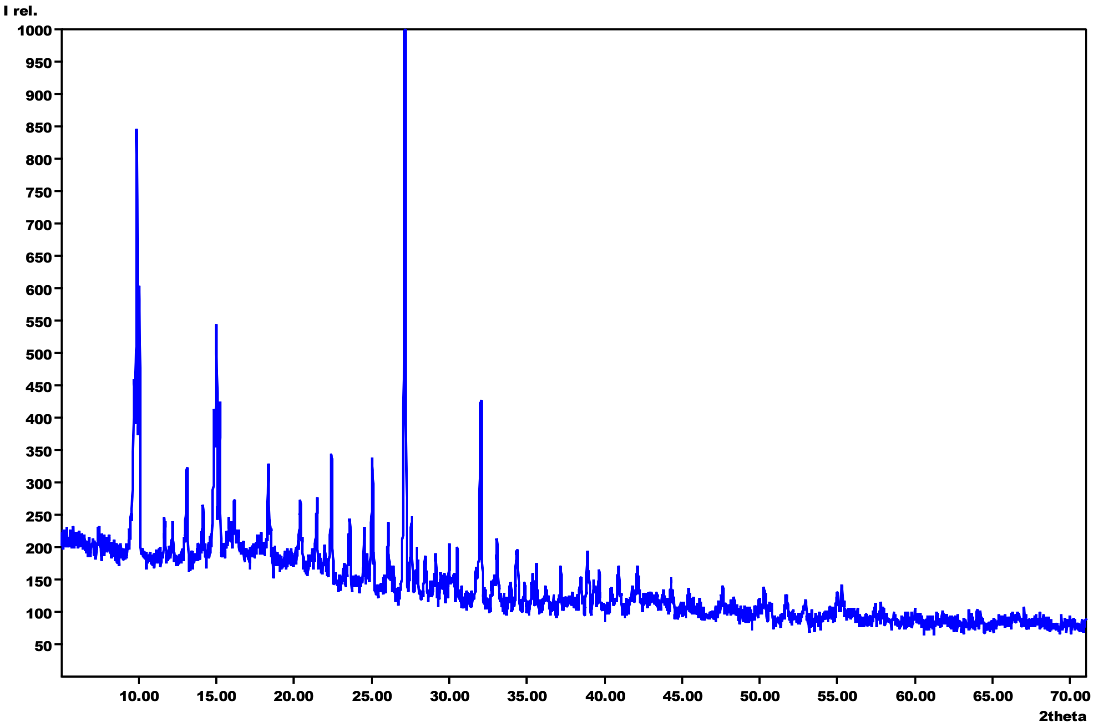

Figure S2. Ex-situ powder XRD pattern of an unknown phase formed by heating a mixture of $MnCl_2$ and melamine in a 1:2 ratio to the first exothermic DSC peak, observed at 306 °C.

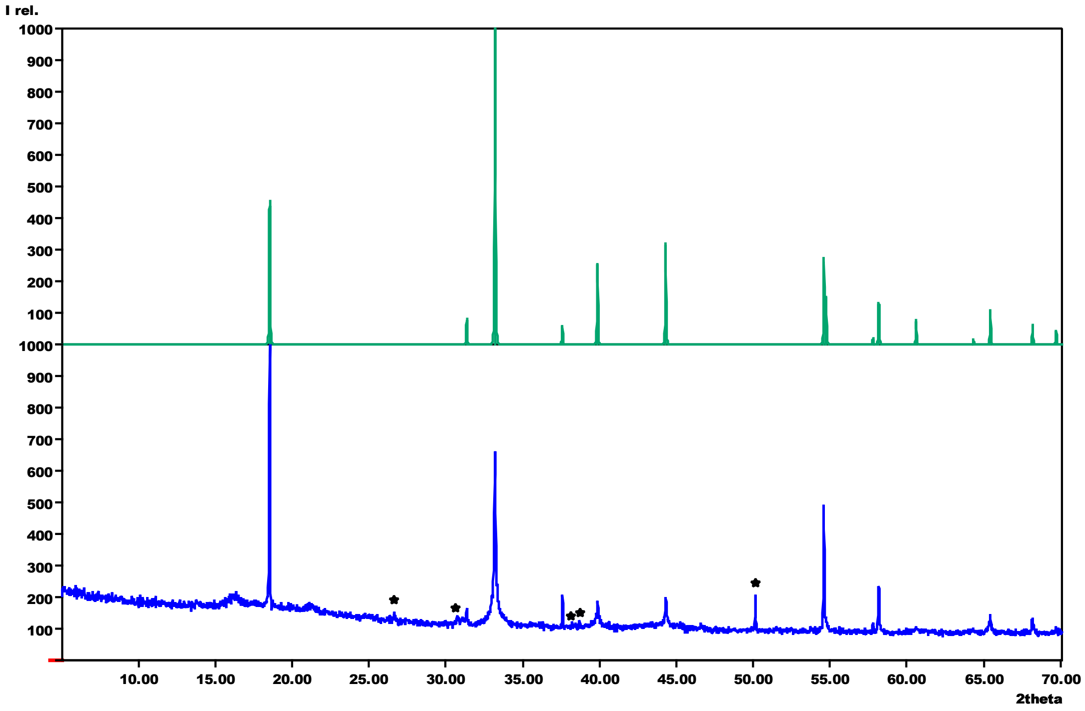

Figure S3. a. XRD pattern of manganese carbodiimide ($MnCN_2$) obtained by heating $MnCl_2(C_6H_6N_{10})$ to 700 °C, along with reflections of unknown side-phase (shown with black stars) compared with the calculated pattern based on the single-crystal structure refinement (top) (CCDC code: 272236). (Please note that the XRD patterns were obtained without annealing the product and only by stopping the decomposition process at 700 ˚C. The high background observed in the patterns can likely be attributed to the fluorescence effect of manganese-containing compounds when using Cu-K_α radiation.)

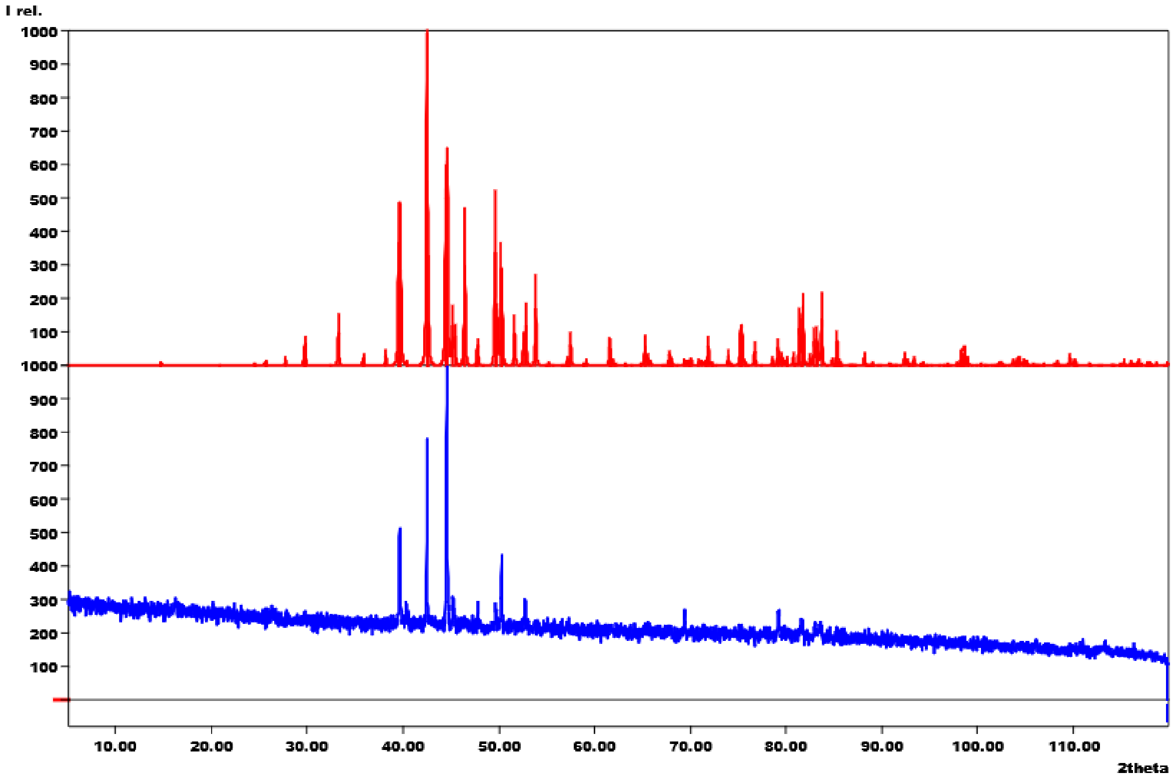

Figure S3.b. XRD pattern of manganese carbide (Mn_7C_3) obtained from heating $MnCl_2(C_6N_{10}H_6)$ to 900 °C (bottom), compared with the calculated pattern based on the single-crystal refinement (top) (CCDC code: 2141509). (Please note that the XRD patterns were obtained without annealing the product and only by stopping the decomposition process at 900 ˚C. The high background observed in the patterns can likely be attributed to the fluorescence effect of manganese-containing compounds when using Cu-K_α radiation.)

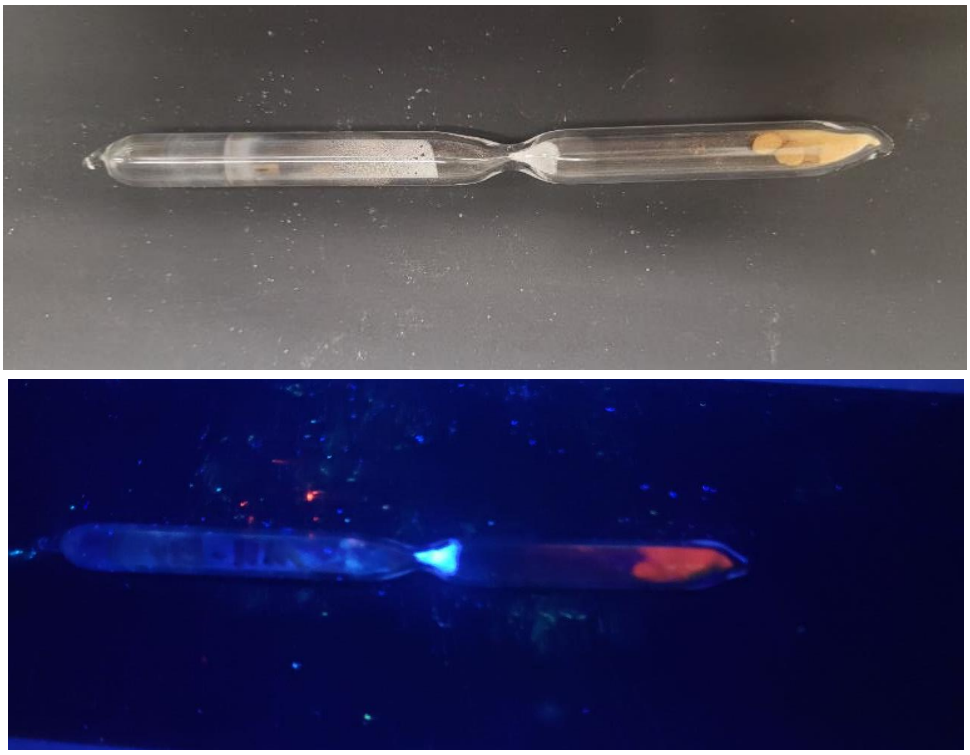

Figure S4a. Photograph of a two-chamber ampule used for separation of side-phase from $MnCl_2(C_6N_{10}H_6)$ under daylight (top) and under UV irradiation (bottom). (The observed blue light observed in the Figure originates from the reflectance of the blue light (366 nm) on the white powder

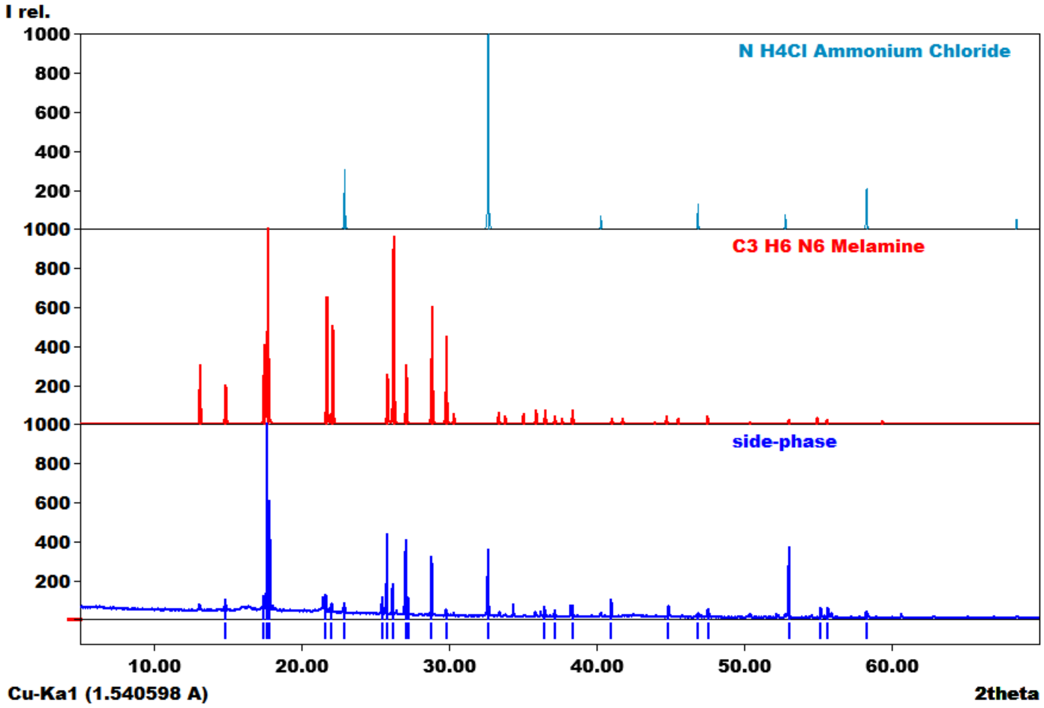

Figure S4b. XRD pattern of side phase on the left side of two-sided chamber.

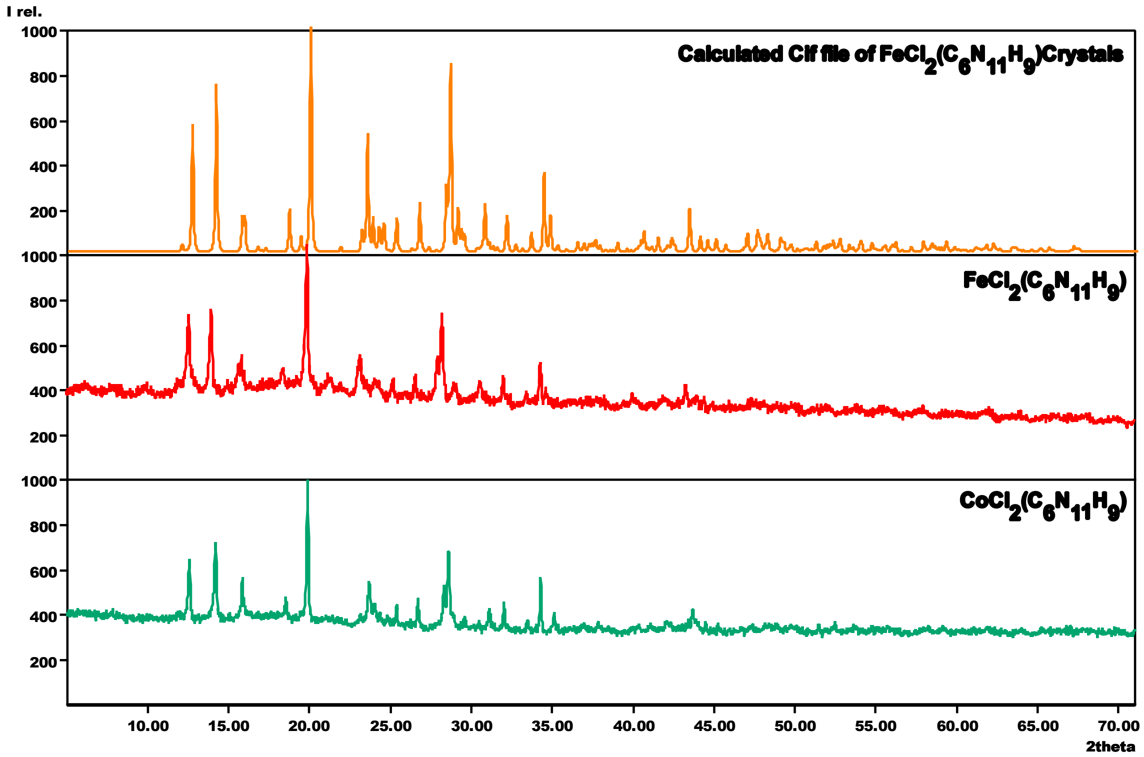

Figure S5. Recorded XRD patterns of $FeCl_2(C_6N_{11}H_9)$, $CoCl_2(C_6N_{11}H_9)$ with the calculated pattern from the structure refinement of $FeCl_2(C_6N_{11}H_9)$ (top).

Table S2. Vibrational frequencies (in cm^{-1}) for $MnCl_2(C_6N_{10}H_6)$ compared to those of melamine and melem.

Vibrational Modes	Melamine	Melem	$MnCl_2(C_6N_{10}H_6)$
**Ring-sextant out-of-plane bending**	813	804	800
**CNC bending**	1193	1306	1313
**Side-chain CN breathing**	1434<br>1440<br>1550	1470	1498
**NH_2 bending**	1652	1612	1610<br>1689
**NH stretching**	3128<br>3334<br>3421<br>3469	3119<br>3325<br>3424<br>3487	3323<br>3431

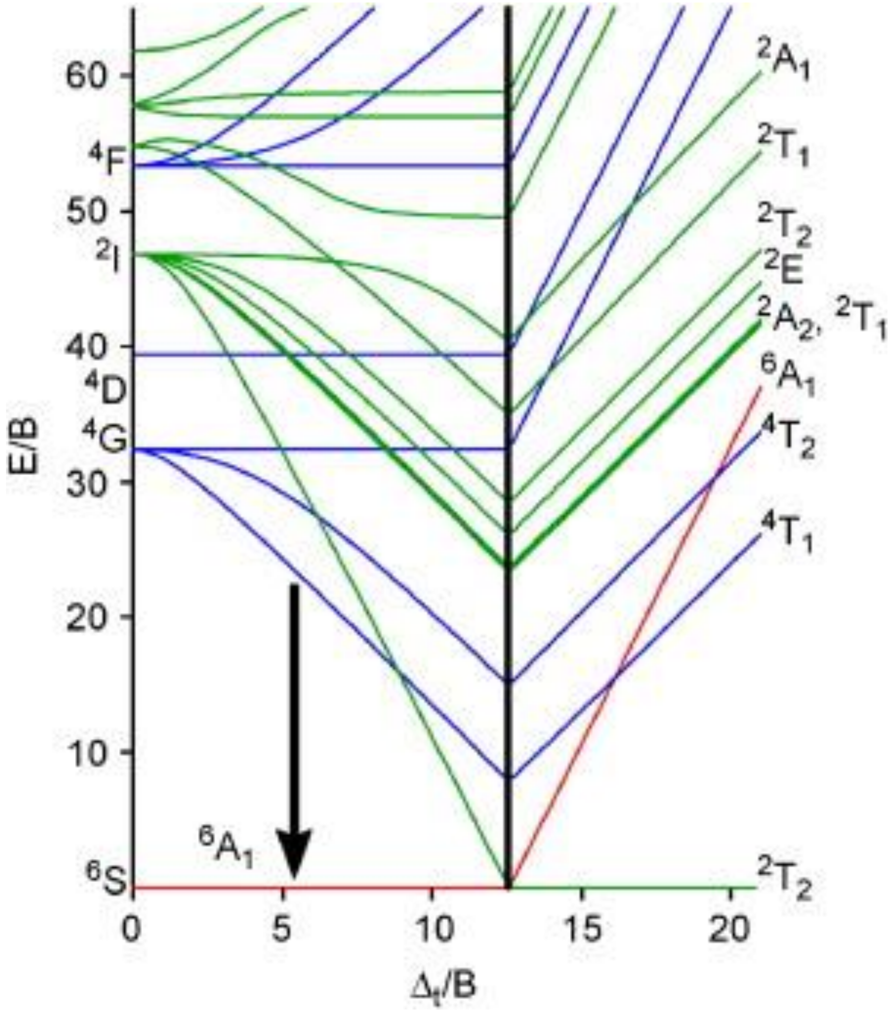

Figure S6. Tanabe-Sugano-Diagram for a d^5 ion with the most prominent emission transition between $^4T_1(^4G)$ and $^6A_1(^6S)$.

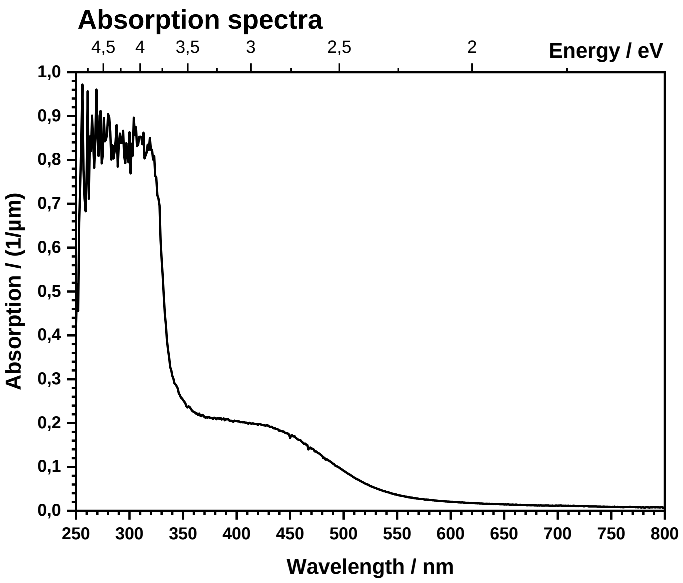

Figure S7. A qualitative absorption spectrum by the aid of the Kubelk-Munk function from the reflection spectrum of $MnCl_2(C_6N_{10}H_6)$.

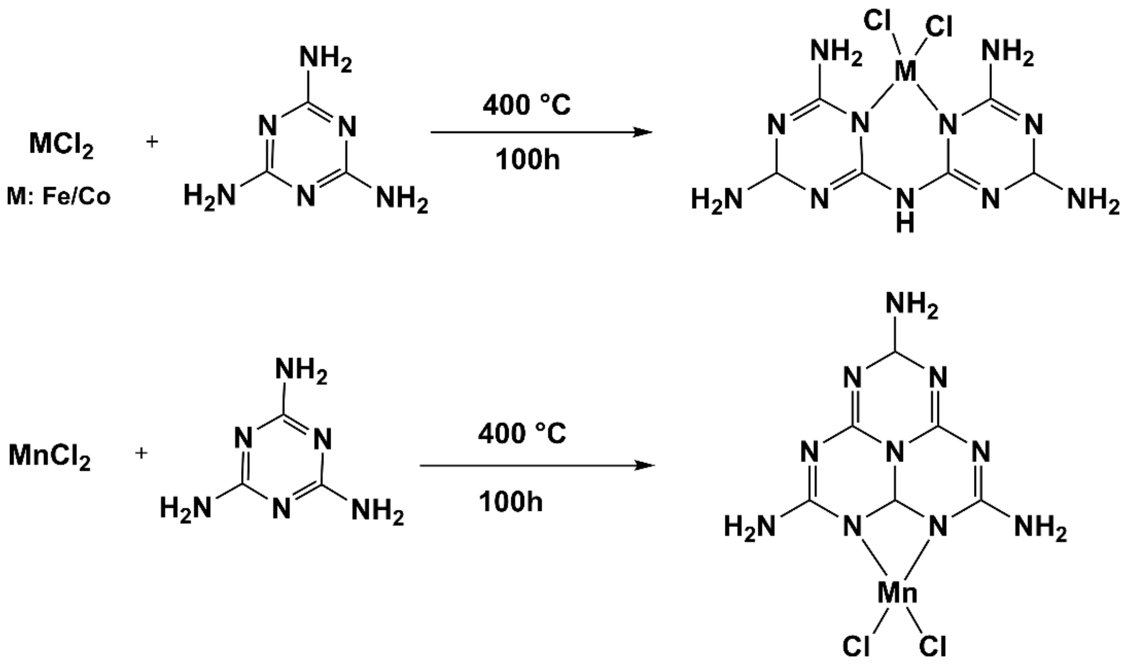

Figure S8. A reaction scheme of melamine with some transition metal chlorides to obtain $FeCl_2(C_6N_{11}H_9)$, $CoCl_2(C_6N_{11}H_9)$, and $MnCl_2(C_6N_{10}H_6)$ compounds
